# Laser microgrooved vs. machined healing abutment disconnection/reconnection: a comparative clinical, radiographical and biochemical study with split-mouth design

**DOI:** 10.1186/s40729-021-00301-6

**Published:** 2021-03-17

**Authors:** Renzo Guarnieri, Gabriele Miccoli, Rodolfo Reda, Alessandro Mazzoni, Dario Di Nardo, Luca Testarelli

**Affiliations:** 1 Department of Oral and Maxillo-Facial Sciences, La Sapienza University of Rome, Rome, Italy; 2 Private Perio-Implant Practice, Strada di Canizzano 33, 31100, Treviso, Italy

**Keywords:** Peri-implant crevicular fluid, Inflammation, Abutments, Cytokines

## Abstract

**Background:**

Repeated removal and replacement of healing abutments result in frequent injuries to the soft tissues.

**Purpose:**

The purpose of this study was to evaluate the effect of disconnection/reconnection of laser microgrooved vs. machined healing and prosthetic abutments on clinical periodontal parameters, marginal bone levels, and proinflammatory cytokine levels around dental implants.

**Material and methods:**

Twenty-four patients each received 2 implants with one-stage protocol in a split-mouth design on the same jaw. In each patient, one healing and prosthetic abutments with a laser microgrooved surface (LMS group) and one healing and prosthetic abutments with machined surface (MS group) were used. Four months following implant placement (T0), the healing abutments were disconnnected and reconnected three times to carry out the impression procedures and metal framework try-in. Four weeks later (T1), definitive prosthetic abutments were installated with screw-retained crowns. Modified plaque index (mPI), modified gingival index (mGI) bleeding on probing (BOP), and probing depth (PD) were recorded at T0 and T1. At the same time points, samples for immunological analyses were taken from the sulcus around each implant. Peri-implant crevicular fluid (PICF) samples were analyzed for interleukin-1beta (IL-1β), interleukin-6 (IL-6), and tumor necrosis factor (TNF)-α levels using the ELISA kit.

**Results:**

At T0 and T1, mPI and mGI showed no statistical difference between the two groups, while higher PD and BoP values were noted for the MS group (*P* < 0.05). The mean PICF volume and mean concentrations of IL-1β, IL-6, and (TNF)-α in the LMS group were statistically less than those in the MS group (*P* < 0.05). In addition, comparison of IL-6 and IL-1β mean concentrations at T0 and T1 in the MS group showed a statistically significant increase (*p* < 0.05) over time, which was not noted for the LMS.

**Conclusion:**

Disconnection/reconnection of healing and prosthetic abutments with a laser-microgrooved surface resulted in less inflammatory molecular response compared with conventional machined ones.

**Trial registration:**

ClinicalTrials.govNCT04415801, registered 03/06/2020

## Introduction

Over the years, some new configurations and topographic characterizations have been proposed and investigated in an attemp to improve the soft tissue attachment to the transmucosal part of dental implants [1–13]. One of the configurations is produced by a controlled laser ablation, which allows the formation of microgrooves with resolution within a micrometric range. *In vitro* experimental studies provided the hypothesis that laser-produced microgrooves in the range of 8 μm could be used to create a predetermined site on which a physical connective tissue attachment can be achieved [14–17]. Evaluating the effect of surface microgeometry in terms of cell attachment, proliferation, and orientation, cell culture studies showed that fibroblasts grown on 8-μm microgrooved surface become oriented and channeled in line with the grooves, whereas on a smooth/machined surface, fibroblasts growth was random. Subsequent histologic studies on humans demonstrated that, unlike fibers aligned in a parallel nonfunctional orientation around dental implants as fibrous capsules, fibers around 8-μm laser-microgrooved surfaces (LMS) have perpendicular, functional physical orientation [18–20]. This kind of attachment is similar to that of a natural tooth, which is an indispensable barrier against bacterial infection and other harmful stimuli. Attachment helps stabilize the peri-implant soft tissue and protect the peri-implant marginal bone. It has been also histologically documented in animal and human models that the peri-implant soft tissue seal developed using an 8-μm LMS on healing abutments can be transferred to another abutment with the same macro and micro-architecture [21–23]. A clinical question, however, remains unanswered: whether repeated removal and reattachment of LMS healing abutments for impression procedures and metal framework try-in disturbs and disrupts the mucosal seal. Repeated disconnection/reconnection of healing abutments with machined surfaces (MS) result in frequent injuries to the soft tissues [24], with subsequent tissue inflammation, apical downgrowth of junctional epithelium, and apical positioning of the connective tissue and crestal bone level changes [25]. To detect peri-implant soft tissue conditions by means of histologic analysis in humans is difficult. Ethical concerns such as harvesting gingival tissue around abutments in humans are major since it may affect long-term healing. A non-invasive method to study the response of inflamed tissues around teeth is the analysis of gingival crevicular fluid (GCF) [26]. In analogy, analysis of peri-implant crevicular fluid (PICF) was proposed [27, 28]. Similar to GCF, the PICF is an osmotically mediated inflammatory exudate which originates from the gingival vessel plexus. The composition of PICF is similar to gingival crevicular fluid, consisting of cells, host-derived enzymes and their inhibitors, inflammatory mediators, host response modifiers, and tissue breakdown products. Clinical studies comparing the inflammatory and immunological responses around implants and teeth found no significant difference between PICF and GCF volumes at either healthy or inflamed sites [27, 28]. Inflammatory and immune events were similar in the peri-implant mucosa and gingiva, and GCF and PICF production was governed by similar mechanisms, which depend on peri-implant tissue inflammatory conditions. Inflammation in peri-implant gingival tissues results in the activation of innate immune receptors, affecting the expression of proinflammatory cytokines which may be detected by analysis of PICF [29–31]. Changes in PICF flow rate and cytokine profiles may occur based on the health status of the peri-implant mucosa and may help in detecting early metabolic and biomechanical lesions in peri-implant soft tissues.

The present study aimed to evaluate, clinically and biochemically, the effect of repeated disconnection/reconnection of  LMS and MS healing abutments during restorative stages, on hard and soft tissue behavior.

## Material and methods

### Patients

Twenty-four partially edentulous patients (11 males and 13 females, mean age 47.3 years), requiring implant therapy for a prosthetic rehabilitation in at least two contralateral sites of the mandible or maxilla, participated in this study. Patients were included if they were 18 years or older, in good general health, with sufficient amount of bone available to place a standard implant (3.8 mm diameter and 9 mm length) detectable by means of CBCT evaluation, and with adequate width of keratinized tissue (≥ 2 mm) at the implant site.

None of the recruited patients had received antibiotics in the last 3 months prior to examination. Exclusion criteria were as follows: natural teeth adjacent to surgical area affected by untreated periodontal or endodontic infections, peri-implant bone defects requiring bone augmentation, absence of opposing occlusion, full-mouth plaque score (FMPS) ≥ 15%, full-mouth bleeding score (FMBS) ≥ 15% recorded at the time of implant placement, para-functional habits, severe maxilla–mandibular space discrepancies, uncontrolled diabetes and treatment with bisphosphonates, patients smoking > 10 cigarettes a day, and any drug/alcohol abuse.

Secondary exclusion criteria at implant surgery included a lack of primary implant stability or the need for a simultaneous hard tissue grafting. Secondary exclusion criteria at end of healing phase included FMPS and FMBS ≥ 15%, clinical signs of implant mobility, or any sign of peri- implant infection.

Each patient was informed about the evidence-based positive outcome of implant treatment and signed a free informed consent form after he/she has received detailed information about the study.

Treatments were performed according to the principles outlined in the Declaration of Helsinki on experimentation involving human subjects. The study was approved by the Research Ethics Committee of the La Sapienza University of Rome (#4597). Trial registration: ClinicalTrials.gov NCT04415801,registered 03/06/2020, https://register.clinicaltrials.gov/prs/app/action/SelectProtocol?sid=S0009V52&selectaction=Edit&uid=U0003LQX&ts=24&cx=-mviyyi.

**Fig. 1:**
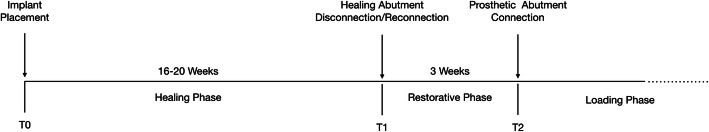
Overview of the study outline and follow-up visits

The study duration was from May 2016 to September 2017, and it was accomplished in 2 phases, including healing period following implant placement (12/16 weeks) and a restorative phase (3 weeks) (Fig. 1).

### Implants

Forty-eight implants (Tapered Internal TRX, BioHorizons, AL, USA) were inserted by the same surgeons (RG, LT) using the same one-stage protocol in accordance with the manufacturer’s recommendations and with surgical procedures suggested for one-stage implant placement in partially edentulous patients.

### Surgical and prosthetic protocol

Following local anesthesia, a crestal incision over the edentulous site was performed. After full-thickness flap elevation, osteotomy site was prepared using custom-made surgical template. After placement, one implant received a healing abutment with LMS (Laser-Lok© Healing Abutments, BioHorizons, AL, USA) (LMS group), while the contralateral implant received a healing abutment with MS (Standard Healing Abutment, BioHorizons, AL, USA) (MS Group). The MS abutments were entirely machined, while the LM abutments exhibited a partially (0.7 mm) microgrooved surface. The resulting circumferential horizontal mismatch (i.e., platform switch) was 0.65 and 0.6 mm for 4.3- and 5.0-mm implant diameters, respectively.

The single-stage approach prevents soft tissue covering the implant and corresponding cover cap during early postoperative period. A minimum distance of 1.5 mm between implant and adjacent teeth was maintained to preserve surrounding soft tissue and bone. Postoperative instructions included antimicrobial rinse (0.12% chlorhexidine rinse) and analgesics. Prescription medication use was recorded through patient diaries. Sutures were removed at week 1.

After 4 months (T0), the healing abutments were removed and placed in a saline solution. After taking the impression with direct pick-up coping and an open-tray, the healing abutments were reattached. The same procedure for removal and reattachment of the same healing abutment was repeated two additional times for the try-in of crown frame and the try-in of crown porcelain. Each time, the inside of the implant and the transmucosal area were rinsed with 0.2% chlorhexidine to eliminate foreign agents that induced inflammation. During the prosthesis delivery session, the laser-microgooved healing abutments were replaced with laser-microgrooved definitive prosthetic abutments (Laser-Lok© Easy Ti Abutments, BioHorizons, AL, USA), and smooth/machined healing abutments were replaced with smooth/machined definitive prosthetic abutments (Custom Castable UCLA Abutments, BioHorizons, AL, USA).

At T1, no rinsing with 0.2% chlorhexidine was performed. All implant-supported restorations were screw-retained single crowns. After delivery of the final restoration, patients were enrolled in a maintenance program with biyearly recalls. In Table 1, demographic data of the study population are reported.

**Table 1:** Demographics and healing/prosthetic abutment data of study population

Patients	24	
Sex (male/female)	11/13	
Age (years)		
Range	24/67	
Mean ± SD	47.3 ± 9.4	
Healing/prosthetic abutment	LSM	MS
Jaw location (maxilla/mandible)	12/16	12/16
Tooth type (molar/premolar)	10/18	10/18
Implant diameter (3.8 mm/4.6 mm)	18/10	20/8
Implant length (9.5 mm/11 mm/12.5 mm)	15/7/6	14/8/6

### Clinical data and sample collection

The clinical evaluation was performed by one clinician (RG). At T0 and T1 (Fig. 1), the following clinical parameters were assessed at each implant site using a pressure-calibrated (20–25 g) and color-coded plastic periodontal probe (Click-Probe® green, Kerr GmbH, Biberach, Germany): modified plaque index (mPI), modified gingival index (mGI), bleeding on probing (BoP), and probing depth (PD). mPI, mGI, BOP, and PD measurements were performed at 6 aspects per implant: mesiobuccal (mb), midbuccal (b), distobuccal (db), mesiooral (mo), midoral (o), and distooral (do). In addition, at T0 and T1, FMPS and FMBS were assessed

### PICF sampling and biochemical analysis

The PICF was collected with standardized paper strips (Periopaper™, Proflow, Amityville, NY) at T1 and T2 by one clinician (RG). Following the isolation of the sampling area with sterile cotton gauzes, accurate suction was performed, and experimental sites were gently air dried to reduce any possible contamination with saliva. Supragingival plaque was removed with teflon or plastic curettes for implant maintenance. Extreme care was taken to minimize mechanical irritation during PICF sampling because this is known to affect the actual fluid volume. Two paper strips were placed at each sampling site at the same time (mesially and distally) and were left *in situ* for 30 s. Paper strips contaminated by blood were excluded. To eliminate the risk of evaporation, paper strips with PICF were immediately transported to previously calibrated Periotron 8000® (Ora Flow, Inc., Plainview, NY, USA) which was switched on and allowed to warm up for volume quantification. Before volume measurement, a blank paper strip was placed in the device, and the reading dial was set to zero. To increase reliability, the calibration of the device was checked periodically by triplicate readings. The PICF was measured electronically in Periotron units, which were converted to microliters (μl) by the MCCONVRT software (Ora Flow).

For the biochemical analysis, the paper strips were placed in a single Eppendorf vial containing 100 μl phosphate-buffered saline and stored at − 80 °C. Interleukins (IL-1β, IL-6) and tumor necrosis factor alpha (TNF-α) were quantified by enzyme-linked immunosorbent assay (ELISA) kits following the procedures recommended by the manufacturer (Duoset kit; R&D,Minneapolis, MN, USA). The standard solution and samples were added to wells, which had been precoated with specific monoclonal capture antibodies. After 3 h, polyclonal antibodies conjugated with horseradish peroxidase were added to each well and incubated for 1 h. A substrate solution containing hydrogen peroxidase and chromogen was added and allowed to react for 20 min. The biomarker levels were assessed by a micro-ELISA reader (Ultramark, Bio-Rad, CA, USA) at 450 nm and normalized to the abundance of standard solution. All biochemical analyses were performed by a blinded researcher.

### Radiograph examination

Radiographs were performed at T0 and T1, with a paralleling technique using a Rinn film holder with a rigid film-object X-ray source. For the radiograph procedure, a silicone index material was fixated to the residual dentition, and a radiograph holder was constructed for each patient. This technique ensured that the same position of the radiograph film could be reproduced at each visit, and the angle of the radiograph would not deviate. The radiographs were taken in high-resolution mode (Vista Scan Durr Dental, Durr Dental Italy S.r.l) with a dental X-ray machine (TM 2002 Planmeca Proline CC, Planmeca Group Helsinki, Finland) equipped with a long tube that operated at 70 Kw/7.5 mA. Specialized software (DBSWIN software, Durr Dental Italy S.r.l) was used for linear measurements of marginal bone changes. The following radiographic measurements were performed:

- radiographic implant length (IL): distance (in mm) between the implant coronal margin and the implant apex as assessed at the midportion of the implant; **and**
- residual bone height at the mesial (MI) and distal (DI) aspects of the implant: distance (in mm) between the line linking the coronal implant margin, and the first contact of the crestal bone on both mesial and distal sides of the implant.

To account for radiographic distortion, radiographic measurements on each radiograph were adjusted for a coefficient derived from the ratio: true length of the implant/IL.

The radiographic marginal bone loss (MBL) was calculated by subtracting the marginal bone level at T0 from marginal bone level at T1.

All measurements were carried out by a single trained examiner (RG) who had previously undergone a calibration session for radiographic assessment on a sample of 5 patients treated with the same implant system and not included in the study.

### Statistical analysis

Due to a lack of published data to estimate the variations in outcome measures at the respective test and control abutments, a sample size calculation was not feasible. Data were analyzed using SPSS software version 13.0 (Chicago, IL, USA).

Mean values and standard deviations (mean ± SD) were calculated for each considered clinical parameter. The normal distribution of error terms and the validity of the model’s assumptions were assessed using a Q–Q plot. One-sided *P* values were calculated and corrected according to Sidak’s method for multiple hypothesis testing. Data were analyzed by means of Mann–Whitney test and by repeated-measure analysis of variance (ANOVA). The Bonferroni test was applied for the pairwise comparison. A *P* value < 0.05 was considered statistically significant. Results of the linear mixed effects were used to calculate the estimates of the differences between groups and their 95% confidence intervals.

### Clinical results

**Fig. 2:**
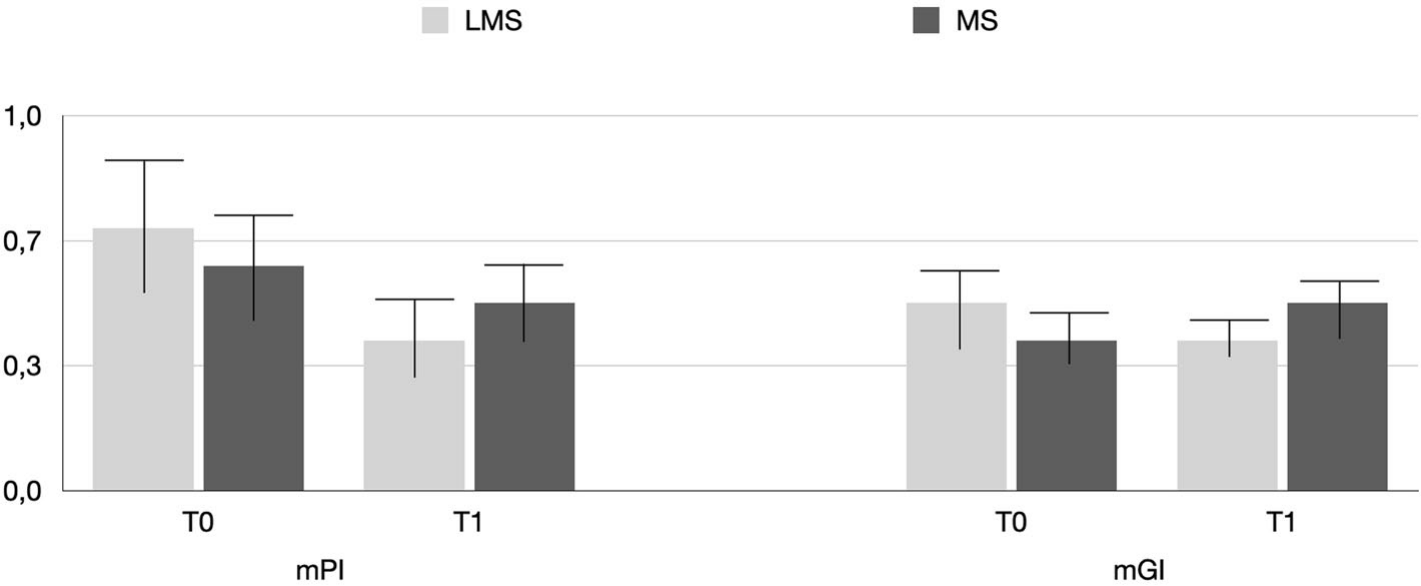
Mean mPI and mGI values recorded around LMS and MS abutments during the study period

**Fig. 3:**
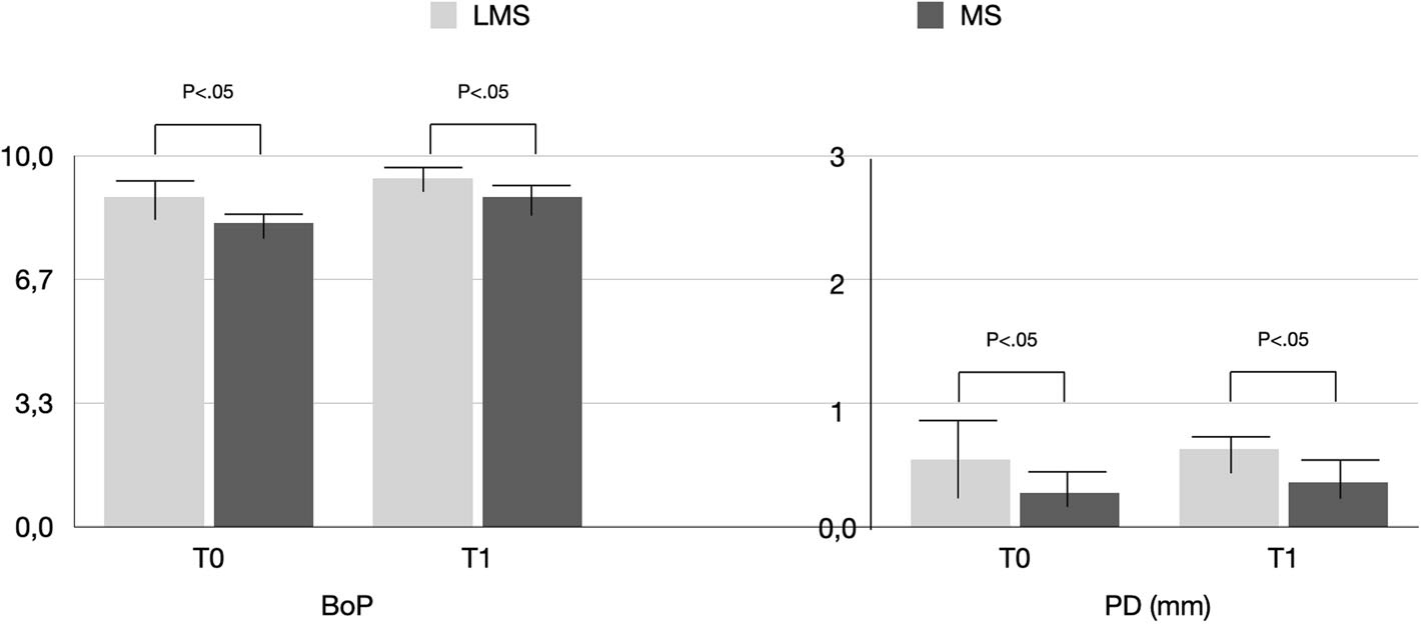
Mean BoP and PD values recorded around LMS and MS abutments during the study period

Table 1 reports the demographics and healing/prosthetic abutments data of the study population. The mPI for MS vs LMS at T0 and T1 were 0.7 ± 0.2 vs. 0.6 ± 0.3 and 0.4 ± 0.2 vs. 0.5 ± 0.1, respectively (Fig. 2). There was no significant difference between T0 and T1 in either group (*P* > 0.05). Similarly, minimal gingival inflammation was noted throughout the study in both groups. At T0 and T1, the mGI for MS was 0.5 ± 0.13 and 0.4 ± 0.21, respectively. At the same time points, the mGI for LM was 0.4 ± 0.14 and 0.5 ± 0.22, respectively. There was no significant difference between T0 and T1 in either group (*P* > 0.05) (Fig. 2). The BoP for LMS vs MS were 7.8 ± 0.2 and 8.9 ± 0.8, respectively at T0 and 8.2 ± 0.7 vs. 9.4 ± 0.4 respectively at T1 (Fig. 3). There was no significant difference between T0 and T1 in either group (*P* > 0.05), while differences between MS and LMS at each time point were statistically significant (*P* < 0.05). Between T0 and T1, a statistically significant increase pattern in the MS group was noted for PD (*P* < 0.05), while in the LMS group, PD showed no statistically significant increase (Fig. 3). In the LMS group, the mean PD at T0 and T1 were 0.9 ± 0.3 mm and 1.1 ± 0.2 mm, respectively. In MS, the mean PD at T0 and T1 were 1.8 ± 0.4 mm and 2.1 ± 0.1 mm, respectively. Difference in PD between the MS and LMS groups was statistically significant at each time point (*P* < 0.05).

### Biochemical results

**Fig 4:**
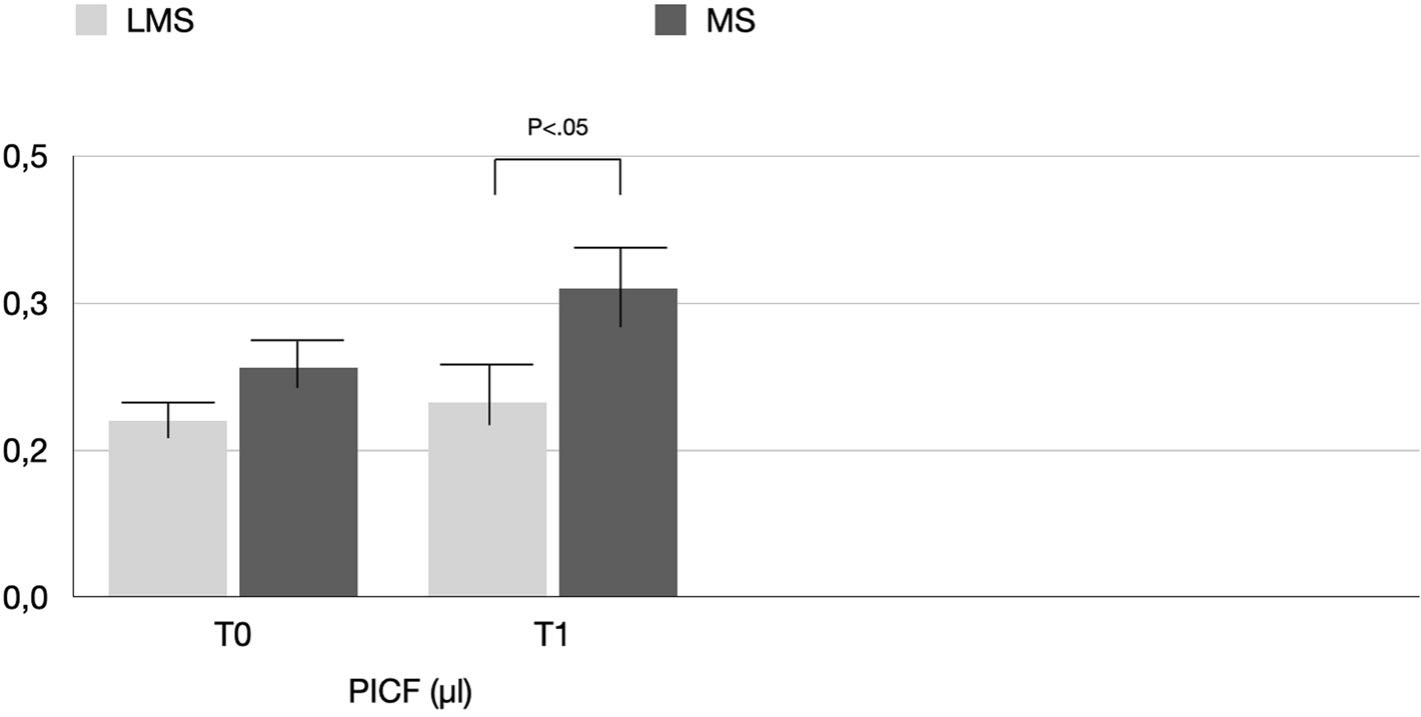
Mean values of PICF (μl) detected in the LMS and MS groups

**Fig. 5:**
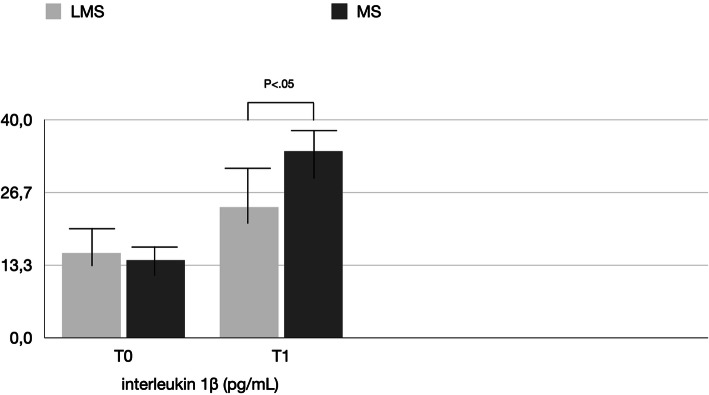
Mean levels of interleukin 1β (pg/mL) detected in the LMS and MS groups

**Fig. 6:**
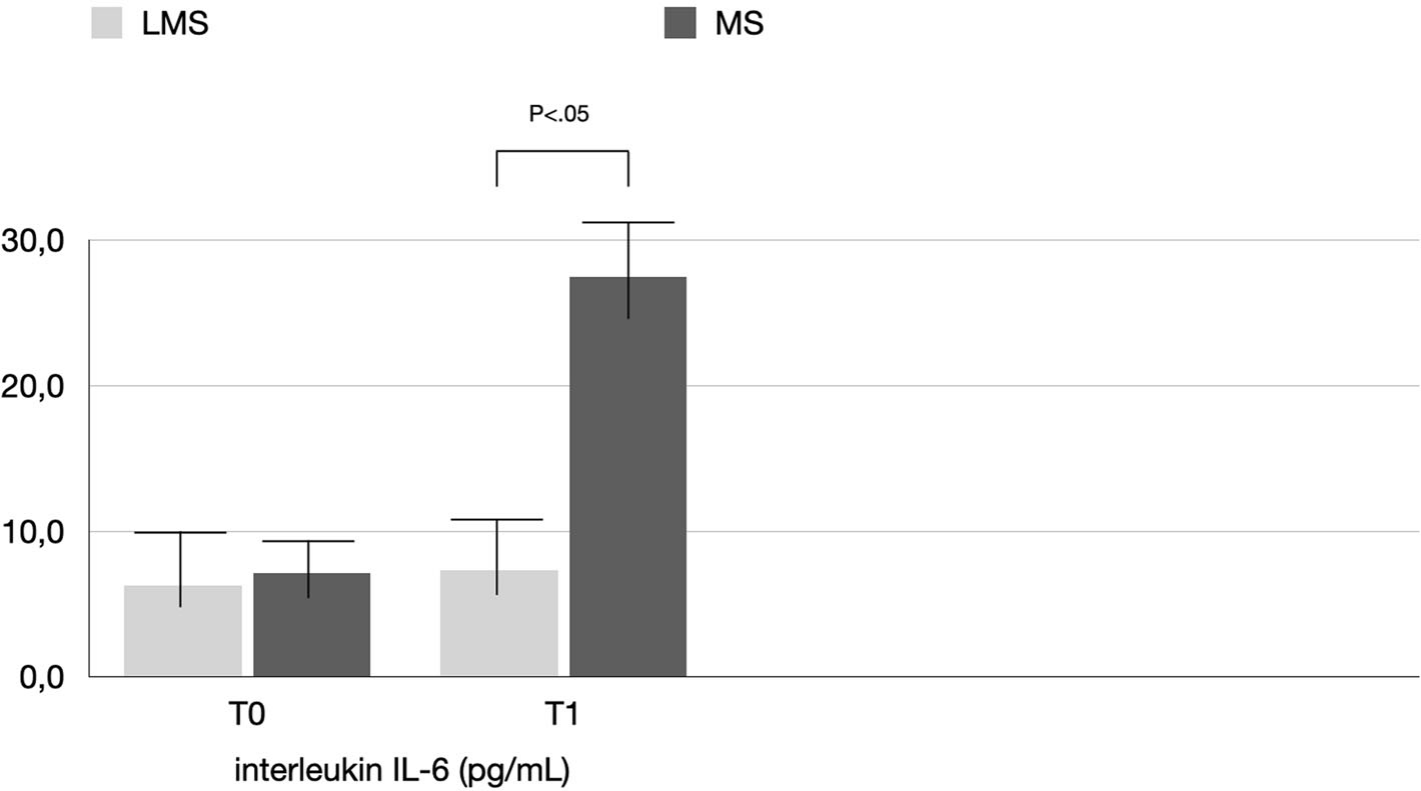
Comparison of the level of interleukin IL-6 (pg/mL) detected in the LMS and MS groups

**Fig. 7:**
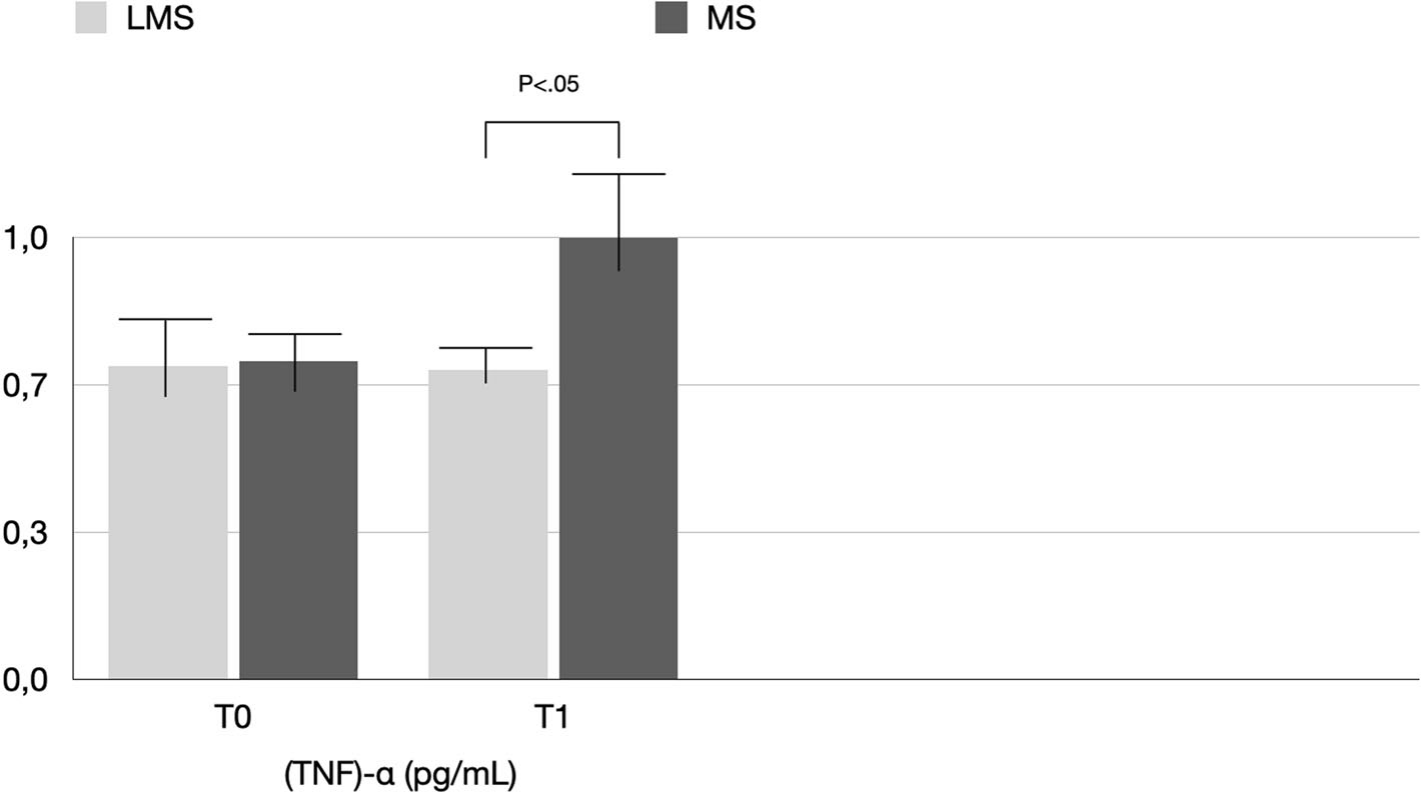
Comparison of the level of tumor necrosis factor (TNF)-α (pg/mL) detected in LMS and MS

PICF total volumes for MS vs LMS were 0.22 ± 1.5 μL vs. 0.20 ± 1.5 μL at BSL, 0.35 ± 0.16 μL vs. 0.26 ± 0.12 μL at T0, and 0.48 ± 0.12 μL vs. 0.24 ± 0.18 μL at T1 (Table 2, Fig. 4). Differences between T0 and T1 were statistically significant only for the MS group. Between-group comparisons revealed significant difference (*P* < 0.05) for the MS and LMS groups at each time point. The biomechanical results are reported in Tables 3, 4, 5 and 6 and shown in Figs. 5, 6, and 7. In the MS group, IL-1β and IL-6 showed a significant difference over time, while TNF-α showed no significant difference between T0 and T1. In the LMS group, IL-1β, IL-6, and TNF-α showed no significant difference between over time. At each time point, a significant difference between the MS and LMS groups was detected for IL-1β and IL-6.

**Table 2:** Comparison of the mean value of PICF (μl) detected in the LMS and MS groups

Time	LMS			MS			
	Minimum	Maximum	Mean ± SD	Minimum	Maximum	Mean ± SD	***P***
**T0**	0.11	0.26	0.20 ± 1.5	0.13	0.27	0.22 ± 1.5	> 0.05
**T1**	0.17	0.22	0.26 ± 0.12	0.38	0.49	0.35 ± 0.16	< 0.05
***P***	> 0.05	> 0.05	> 0.05	< 0.05	< 0.05	< 0.05

**Table 3:** Comparison of the level of interleukin 1β (pg/mL) detected in the LMS and MS groups

Time	LMS			MS			
	Minimum	Maximum	Mean ± SD	Minimum	Maximum	Mean ± SD	***P***
**T0**	1.52	23.52	15.53 ± 5.5	1.47	26.12	23.95 ± 7.5	> 0.05
**T1**	1.61	26.27	14.2 ± 4.1	11.71	38.27	34.2 ± 8.1	< 0.05
***P***	> 0.05	> 0.05	> 0.05	< 0.05	< 0.05	< 0.05

**Table 4:** Comparison of the level of interleukin IL-6 (pg/mL) detected in the LMS and MS groups

Time	LMS			MS			
	Minimum	Maximum	Mean ± SD	Minimum	Maximum	Mean ± SD	***P***
**T0**	0.35	12.82	6.24 ± 4.5	0.31	15.73	7.33 ± 2.6	> 0.05
**T1**	0.42	13.51	7.12 ± 4.1	0.74	43.54	27.48 ± 5.9	< 0.05
***P***	> 0.05	> 0.05	> 0.05	< 0.05	< 0.05	< 0.05

**Table 5:** Comparison of the level of tumor necrosis factor (TNF)-α (pg/mL) detected in the LMS and MS groups

Time	LMS			MS			
	Minimum	Maximum	Mean ± SD	Minimum	Maximum	Mean ± SD	***P***
**T0**	0.62	1.12	0.71 ± 0.26	0.57	1.25	0.70 ± 0.14	> 0.05
**T1**	0.63	1.13	0.72 ± 0.21	0.69	1.31	0.73 ± 0.38	> 0.05
***P***	> 0.05	> 0.05	> 0.05	< 0.05	< 0.05	< 0.05

**Table 6:** Peri-implant radiographic bone levels (mm) detected in the LMS and MS groups

Time	LMS	MS	***P***
	Mean ± SD	Mean ± SD	Δ
**T0**	0.11 ± 0.26	0.28 ± 0.14	< 0.05
**T1**	0.36 ± 0.11	0.64 ± 0.18	< 0.05
**Δ**	0.25 ± 0.17	0.36 ± 0.06	< 0.05

### Radiographic results

Between T0 and T1, in the MS group the mean radiographic MBL was 0.36 ± 0.06 mm, while in the LMS group, it was 0.25 ± 0.17 mm. The difference between the MS and LMS groups was noted to be statistically significant (*P* < 0.05) (Table 6).

## Discussion

Before final prosthesis fabrication, healing abutments must be disconnected and reconnected several times for impression making, metal framework try-in, and delivery of definitive abutments. It has been histologically documented that the dis/reconnected manipulation of healing abutment may mechanically injure the soft tissue barrier and disrupt the established mucosal seal, leading to an apical shift of the connective tissue attachment and remodelling of the underlying bone. Frequently, these histological changes clinically result in apical repositioning of the soft tissues [25, 32].

In the present study, the one-stage surgical protocol, with immediate placement of healing abutments, allowed optimal soft tissue access to evaluate both the healing period following implant placement (12/16 weeks) and both the restorative phases (3 weeks). It is known that the peri-implant soft tissue clinical maturity is established 4 weeks following implant placement by a one-stage surgical protocol [33]. Moreover, it has also been documented that the type of peri-implant tissue organization during soft tissue clinical maturity is influenced by transmucosal implant geometry and surface. While a smooth two-dimensional surface may lead to a flat arrangement of cell attachments and a consequent extensive spreading and de-differentiation [34, 35], a three-dimensional roughened or bio-activated surface, conversely, was demonstrated to induce cell differentiation, according to the so-called “contact guidance” concept [36]. During the soft tissue healing phase following implant placement, microgeometric or biofunctional surface modifications may induce fibroblast stabilization and differentiation, which promote connective tissue adaptation to the transmucosal part of the implant. In the present study, at the end of the healing period following implant placement, a significant statistical difference in PD and BoP was found between LMS vs MS. Therefore, it is possible to assume that during the observation periods, surfaces created with a laser on healing abutments may have influenced the soft tissue healing, improving soft tissue adhesion and creating a more robust perpendicular collagen fiber attachment [18–21].

Better results in PD and BoP around LMS vs. MS abutments were found also in a recent study by Schwarz et al. [37]. Authors evaluated clinically, biochemically, and microbiologically the response of the peri-implant soft tissue around LMS and MS abutments during a wound healing period following implant placement (12 weeks), a plaque exposure phase (21 days), and a resolution phase (16 weeks). At the end of the wound healing period, the 46.2% of the MS and merely 13.3% of the LMS abutments revealed a positive BoP. Moreover, the mean BoP tended to be higher at MS compared with LMS abutments, even if the difference did not reach statistical significance. As regards the PD, at the end of the wound healing period, MS and LMS abutments showed a similar mean PD value; at the end of the plaque exposure and resolution phases in the LMS group, the PD was reduced on average by 0.15 mm, while in the MS group, it increased on average by 0.8 mm.

Histological studies in animals and humans provided evidence of reattachment of the connective tissue when a laser-microgrooved healing abutment was replaced with another laser-microgrooved healing or definitive abutment [19, 21, 23]. In this context, in the MS group of the current study, PD showed a statistically significant increase during the restorative phase (between T0 and T1), while in the LMS group, it remained mostly constant. The time interval between T0 and T1 corresponds with prosthetic procedures (impression, proving of crown frame and proving of crown porcelain) during which replacement of the healing abutment was repeated at least three times. Therefore, it is possible to assume that the lower mean values of PD and BoP recorded in the current study at the end of the restorative phase (T1) in the LMS group may be connected to different response of peri-implant gingival tissues to repeated disconnection/reconnection of LMS and MS abutments. It must be emphasized that at each visit, a professionally administered plaque removal at the respective implants and remaining teeth was provided in each patient, who had previously been instructed about suitable oral hygiene and effective plaque control around the healing abutment. This may have undone the possible susceptibility of LMS surfaces to an undisturbed plaque formation, reported in the study by Schwarz et al. [37] as the cause of the higher incidence of diseased LMS vs. MS implants noted at the end of the plaque exposure phase (21 days). The observations of the present study basically corroborates the findings of previous clinical studies which pointed out that in patients enrolled in a professional, strict controlled oral hygiene regimen, LMS implants, compared with implants without LMS, are not more vulnerable to pathogenic microflora colonization [38, 39].

When further analyzing the present data, it was also noted that multiple abutment disconnections/reconnections could have a modest effect on marginal bone level changes regardless of the type of surface of the abutments. In this regard, LMS showed less vertical bone loss (0.11 mm), but the difference is of slight clinical significance. The reason for the slight difference may be that the hard tissue change around the implant is a very complex process which takes longer to manifest than those used in the current study, and that the abutment manipulation is not the only factor to affect the change.

Changes in PICF flow rate and IL-1, IL-1β, and TNF-α profiles evaluated during the current study allowed to get specific information on early metabolic and biomechanical responses around LMS and MS abutments [27, 31, 40]. The higher mean values of PICF flow rate and IL-6 and IL-1β levels recorded around MS abutments at all time points suggests a worsening in peri-implant soft tissue health, that coincides with the higher clinical inflammatory scores recorded around MS abutments compared with LMS. Moreover, in the MS group, PICF flow rate and IL-6 and IL-1β levels increased significantly during the restorative phase (between T0 and T1), while in the LMS group at the same time points, levels remained almost constant. It has been documented that the volumes of PICF increases as a function of greater vascular permeability and ulceration of the epithelium at inflamed sites and that it is directly related to the severity of the tissue inflammation. IL-6 and IL-1β are reliable markers of injury severity in the acute inflammatory response [41–44], and their principal role in gingival tissue is facilitating the healing process by protecting an open wound from bacterial infiltrate [45]. Accordingly, their prolonged production may reflect the extent of tissue trauma and delayed wound healing [46].

A limitation of the current study may be the short follow-up (4 weeks) which does not allow to compare the intensity of the cellular events in peri-implant tissues after implant loading.

Based on the clinical and biochemical results of the current study, it is possible to hypothesize that multiple abutment disconnections/reconnections lead to a different molecular response of peri-implant tissue around LMS and MS, probably connected to different aspects of peri-implant tissue anatomy and physiology (e.g., healing processes). However additional studies with histological analysis and longer follow-up are necessary to confirm these results.

## Conclusion

Within the limits of the present study, it is possible to conclude that multiple disconnections/reconnections of LMS healing abutments resulted in less inflammation compared to conventional MS abutments. The advantage is substantial when repeated removal and replacement of abutments is necessary in the restorative phase.

## Data Availability

The datasets used and analyzed during the current study are available from the corresponding author on reasonable request.

## Abbreviations

LMS: Laser microgrooved surface
SM: Machined surface
T0: Time zero (definitive prosthetic abutment installation)
T1: Time 1 (1 year after functional loading)
PICF: Peri-implant crevicular fluid
IL-1β: Interleukin-1beta
IL-6: Interleukin-6
TNF-α: Tumor necrosis factor alfa
ELISA: Enzyme-linked immunosorbent assay
PD: Probing depth
MBL: Marginal bone loss
mPI: Modified plaque index
mSBI: Modified sulcular bleeding index
BoP: Bleeding on probing
GCF: Gingival crevicular fluid
CBCT: Cone beam computed tomography
FMPS: Full-mouth plaque score
FMBS: Full-mouth bleeding score
RG: Renzo Guarnieri
LT: Luca Testarelli
Kw: Kilowatt
mA: Milliampere
IL: Implant length
MI: Mesial implant aspect
DI: Distal implant aspect
